# Mild autonomous cortisol secretion leads to reduced volumetric BMD at lumbar spine in patients with primary aldosteronism

**DOI:** 10.3389/fendo.2024.1521680

**Published:** 2024-12-12

**Authors:** Nabeel Mansour, Denise Bruedgam, Daniel Heinrich, Ulrich Dischinger, Nicole Reisch, Friederike Völter, Isabel Stüfchen, Elisabeth Nowak, Stephanie Zopp, Victoriya Vasileva, Osman Öcal, Moritz Wildgruber, Max Seidensticker, Jens Ricke, Martin Bidlingmaier, Martin Reincke, Juínia Ribeiro de Oliveira Longo Schweizer

**Affiliations:** ^1^ Department of Radiology, University Hospital, LMU Munich, Munich, Germany; ^2^ Department of Medicine IV, LMU University Hospital, LMU Munich, Munich, Germany; ^3^ Department of Internal Medicine I, Division of Endocrinology and Diabetes, University Hospital, University of Würzburg, Würzburg, Germany; ^4^ Department of Nuclear Medicine, University Hospital of Munich, LMU Munich, Munich, Germany

**Keywords:** MACs, Conn’s syndrome, primary aldosteronism, volumetric bone mineral density, bone turnover markers, cortisol, ACTH

## Abstract

**Objectives:**

Glucocorticoid cosecretion is more common in primary aldosteronism (PA) than previously thought. Chronic subtle cortisol excess in patients with mild autonomous cortisol secretion (MACS) negatively affects bone health. This study aimed to evaluate the impact of MACS on bone density and turnover markers in PA patients.

**Methods:**

Patients with PA and MACS (n = 50), confirmed by a 1-mg dexamethasone suppression test (DST) with a cortisol cutoff of ≥1.8 µg/dL without symptoms of overt Cushing, were compared to age- and sex-matched patients with PA without MACS (non-MACS, n = 50). Lumbar volumetric bone mineral density (vBMD) was extracted by a novel convolutional neural network (CNN)-based framework (SpineQ software v1.0) applied to routine CT data, incorporated into the diagnostic protocol for PA. Additionally, bone turnover markers—including osteocalcin, bone-specific alkaline phosphatase, N-terminal propeptide of type I collagen, and carboxy-terminal crosslinked telopeptide of type I collagen were evaluated between the groups.

**Results:**

Median cortisol after DST was 1.1 µg/dL (30.3 nmol/L) [IQR: 0.5 µg/dL (13.8 nmol/L)] in the non-MACS group and 2.5 µg/dL (69.0 nmol/L) [IQR: 1.4 µg/dL (38.5 nmol/L)] in the MACS group (p < 0.001). Patients with MACS had significantly lower vBMD values compared to the non-MACS group (106.4 mg/cm³ vs. 116.6 mg/cm³, p = 0.038). Cortisol after DST negatively correlated with vBMD (Spearman’s r=−0.33, p=0.00042). No significant differences in bone turnover markers were found, and classifications based on visible lesions on CT or PA-lateralization via adrenal venous sampling did not reveal any significant differences in these markers (p > 0.05 for all comparisons).

**Conclusion:**

Despite non-significant differences in bone turnover markers between patients with PA with or without MACS, CT scans revealed significantly reduced vBMD in PA and MACS patients, indicating compromised bone health and vBMD significantly negatively correlated with cortisol post DST. Thus, opportunistic evaluation of vBMD in routine CT screenings could aid in the early detection of bone alterations in MACS and help mitigate potential long-term adverse effects on bone health.

## 1 Introduction

Primary aldosteronism (PA) is the leading cause of endocrine hypertension (1). Patients with PA typically exhibit hypertension that remains resistant to antihypertensive therapy, which is attributed to excessive secretion of aldosterone. Recent studies have demonstrated a broader impact of PA on metabolism than previously suggested, including impaired insulin secretion and sensitivity, as well as type 2 diabetes mellitus (T2DM) (2–4). Furthermore, large multicenter cohort studies revealed that glucocorticoid cosecretion is a much more prevalent phenotype in PA than previously assumed (5, 6). Recent studies report that mild autonomous cortisol secretion (MACS) in patients with PA, defined as inadequate cortisol suppression following dexamethasone suppression test (DST) (cortisol > 1.8 µg/dL), has an estimated prevalence between 5 and 21% (7, 8). Patients with adrenal incidentalomas and MACS display elevated levels of serum cortisol without the specific clinical manifestations of overt Cushing’s syndrome, such as skin fragility or myopathy (9–11). However, MACS is linked to cardiovascular disease, fragility fractures, frailty and higher mortality rates (11). Therefore, early identification of MACS in patients with PA is of clinical importance. Moreover, adrenalectomy in patients with PA and MACS could potentially lead to a higher risk of adrenal crises if cortisol cosecretion is not detected before adrenalectomy (ADX) and an adrenal insufficiency occurs (12). Adrenal insufficiency has been reported in up to 27% of patients with PA after ADX, and although adrenal crises are rare, they can be life-threatening (13). Therefore, diagnosing cortisol cosecretion is important to assess an additional risk.

Chronic exposure to subtle cortisol excess in patients with MACS has been reported to have negative effects on bone health (14). The prevalence of vertebral fractures in patients with MACS is four times higher than in those with nonfunctioning adrenal tumors (NFAT) (15–17). Furthermore, some studies report that patients with MACS might exhibit abnormal bone metabolism, including low circulating concentrations of bone formation biomarkers, such as osteocalcin (OC) (18) and elevated levels of carboxy-terminal telopeptide of type 1 collagen (CTX-I), indicating increased bone resorption (18–20). However, the exact mechanisms that result in potential bone alterations in patients with PA and MACS are not completely understood.

This exploratory study investigated the effects of prevalent MACS on bone turnover markers and bone density in patients with PA, drawn from the German Conn’s Registry. These patients were compared to age- and sex-matched patients with PA without MACS (non-MACS), as determined by the DST. We hypothesized that due to the known metabolic effects of cortisol (21), patients with PA and concomitant autonomous cortisol secretion may have detectable differences in bone metabolism as shown in imaging and bone turnover markers compared to patients without MACS.

## 2 Materials and methods

### 2.1 Patients

Out of the 269 patients with diagnosed PA included in the German Conn’s registry between the years 2013 and 2022 with non-contrast enhanced CT-imaging, we identified 50 patients from two centers with confirmed MACS (MACS group) (University Hospital of the Ludwig Maximilians University in Munich (LMU) (n = 39) and University Hospital of the University of Würzburg (n = 11) based on a cortisol cut-off value equal to or greater than 1.8 µg/dL post-DST. They were subsequently matched by age and sex with 50 patients with excluded cortisol cosecretion (non-MACS group) (total of n = 100 subjects). Medical records were reviewed for clinical information pertinent to the presentation, laboratory measurements, and therapy, including prevalence of cardiovascular diseases, history of insufficiency fractures, T2DM, and vitamin D, calcium supplementation, antiresorptive therapy, as well as the use of estrogen and antiepileptic medications. The protocol of the German Conn’s Registry was approved by the Ethics Committee of the Medical Faculty at Ludwig Maximilians University Munich (Project number: 24-0273). All patients gave written informed consent.

### 2.2 Clinical and biochemical data

The procedures for PA diagnosis were performed according to the Endocrine Society Practice Guidelines after positive confirmatory testing either through saline infusion or captopril test (22). The absolute aldosterone cut-off value for diagnosing PA was 60 pg/ml (166.5 pmol/l) following recommended cut-off from Endocrine Society and internal validation analysis (23). According to the guidelines, antihypertensive medication was withdrawn or adapted before testing. Confirmatory testing was foregone in cases where aldosterone concentrations were above 200 pg/ml (554.9 pmol/l) with spontaneous hypokalemia and non-detectable renin concentrations (< 2 mU/l or 0.0284 pmol/l). Adrenal venous sampling (AVS) was performed to lateralize the source of aldosterone overproduction (23).

A single DST was performed on all patients at the baseline visit to test for hypercortisolism. Autonomous cortisol secretion was confirmed when cortisol following the DST was equal to or greater than 1.8 µg/dL (49.7 nmol/l). In the case of elevated DST, further tests were performed to exclude overt Cushing syndrome or an ACTH-dependent Cushing (ACTH, 24-h urinary free cortisol or midnight salivary cortisol). Values were determined according to the practice guidelines of the Endocrine Society for diagnosing Cushing’s syndrome, as well as the recently updated guidelines of the Pituitary Society (24, 25). Serum cortisol levels were measured using commercially available chemiluminescence immunoassays (Munich: Liaison; DiaSorin, Sallugia, Italy; Würzburg: Immulite, Siemens Healthineers, Forchheim, Germany). The intra-assay and inter-assay coefficients of variation (CV) were < 10%, with a lower limit of quantification at 0.2 µg/dL (5.5 nmol/l) for both assays. Aldosterone was measured in EDTA plasma using chemiluminescence immunoassays (Munich: Liaison; DiaSorin; Würzburg: IDS-iSYS, Immunodiagnostic Systems, Boldon, UK). Body mass index (BMI) was calculated as body weight in kilograms divided by height in square meters.

### 2.3 Analyses of bone turnover markers

**Figure 1:**
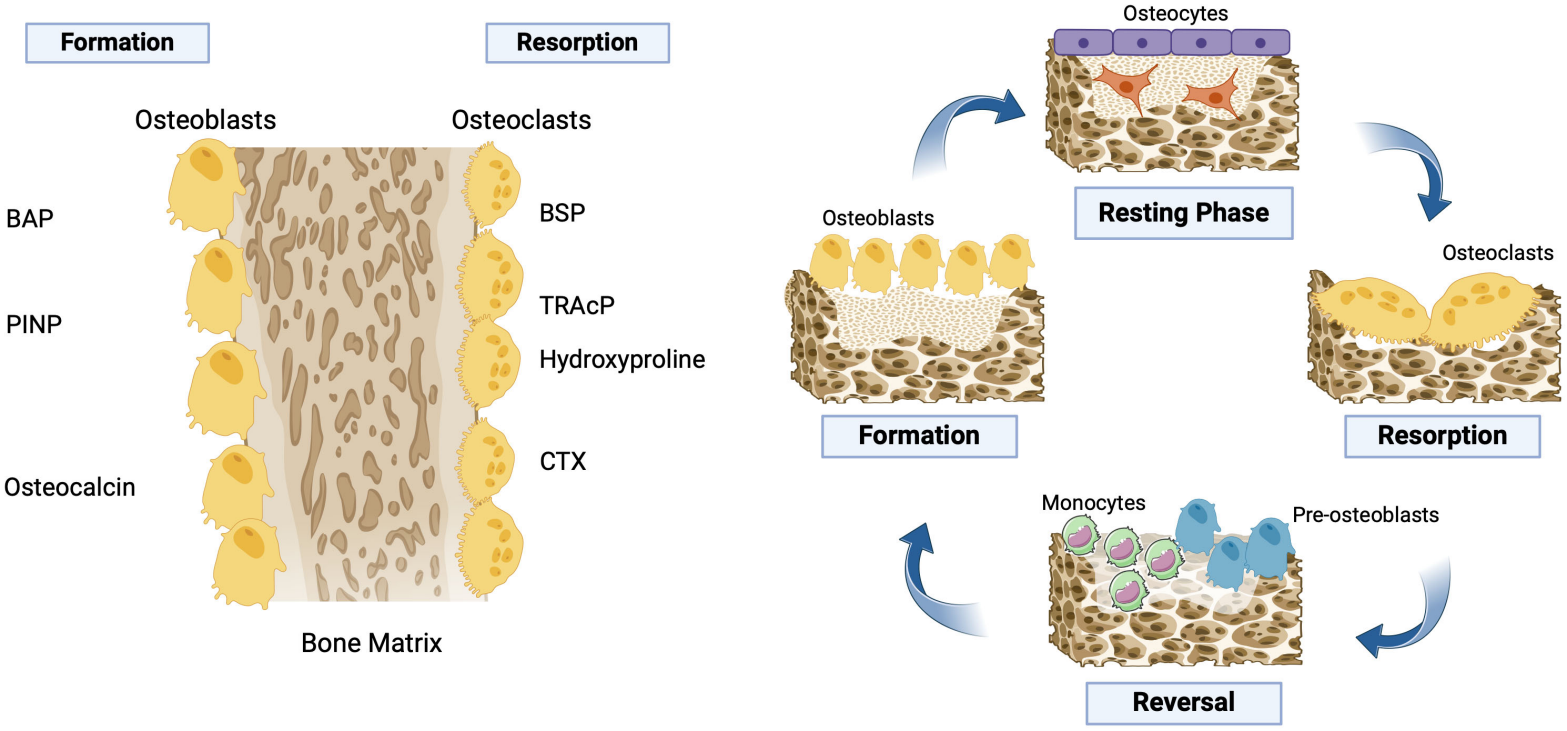
Bone remodeling cycle. This remodeling cycle involves bone “resorption” by the osteoclasts. The osteoclasts remove the old stressed or worn-out mineralized bone. This recreates a “resorption pit.” The “resorption” process causes osteoblasts to become attracted to the “resorption pit.” Osteoblasts rebuild new bone tissue by laying down an unmineralized matrix, called osteoid, which will eventually form new mineralized bone. When this rebuilding is complete, the area of bone remodeling rests until the next remodeling cycle begins. Under normal conditions, the resorption (osteoclast) phase takes approximately 10 days, which is then followed by a formation (osteoblast) phase that can last for up to 3 months. BAP, bone-specific alkaline phosphatase; BSP, bone sialoprotein; CTX, C-terminal telopeptide of type I collagen; PINP, N-terminal propeptide of type 1 collagen; TRAcP, tartrate-resistant acid phosphatase. Created with BioRender.com.

Markers of bone formation (OC), bone-specific alkaline phosphatase (BAP), and N-terminal propeptide of type 1 collagen (PINP) and bone resorption (CTX-I) were analyzed at the Endocrine Laboratory of the LMU (**Figure 1**).

All bone biomarkers were measured in serum samples that were previously stored at -20 ^ο^C. OC, PINP, CTX-I, and Vitamin D were measured using commercially available chemiluminescence immunoassays, while BAP was measured by spectrophotometry (Immunodiagnostic Systems Limited [IDS-iSYS], Frankfurt, Germany) according to the respective kit instructions. The intra-assay and inter-assay coefficients of variation (CV) were 2.5% and 6.0% for OC, 2.9% and 4.6% for PINP, 3.2% and 6.2% for CTX-I, 3.9% and 7.5% for Vitamin D, and 1.6% and 7.3% for BAP, respectively. Parameters related to calcium metabolism were measured using standard methods at the Central Laboratory of LMU.

### 2.4 Adrenal venous sampling

In all cases, adrenal venous sampling (AVS) was performed sequentially without adrenocorticotropic hormone stimulation. During AVS, both plasma cortisol and plasma aldosterone concentrations (PAC) were measured in blood selectively collected from the adrenal veins and simultaneously from the inferior vena cava (IVC). To evaluate the success of adrenal vein catheterization, the selectivity index (SI) was defined as the ratio of plasma cortisol concentration in each adrenal vein to that in the IVC (26, 27). The lateralization index (LI) was defined as the ratio of the aldosterone to cortisol ratio (ACR) on the dominant side with excess aldosterone secretion to the ACR on the non-dominant side. A lateralization index for AVS greater than 4.0, or a lateralization index between 3.0 and 4.0 combined with a contralateral index below 1.0, was considered compatible with unilateral disease. Otherwise, bilateral disease was diagnosed.

### 2.5 Measurement of volumetric bone mineral density

**Figure 2:**
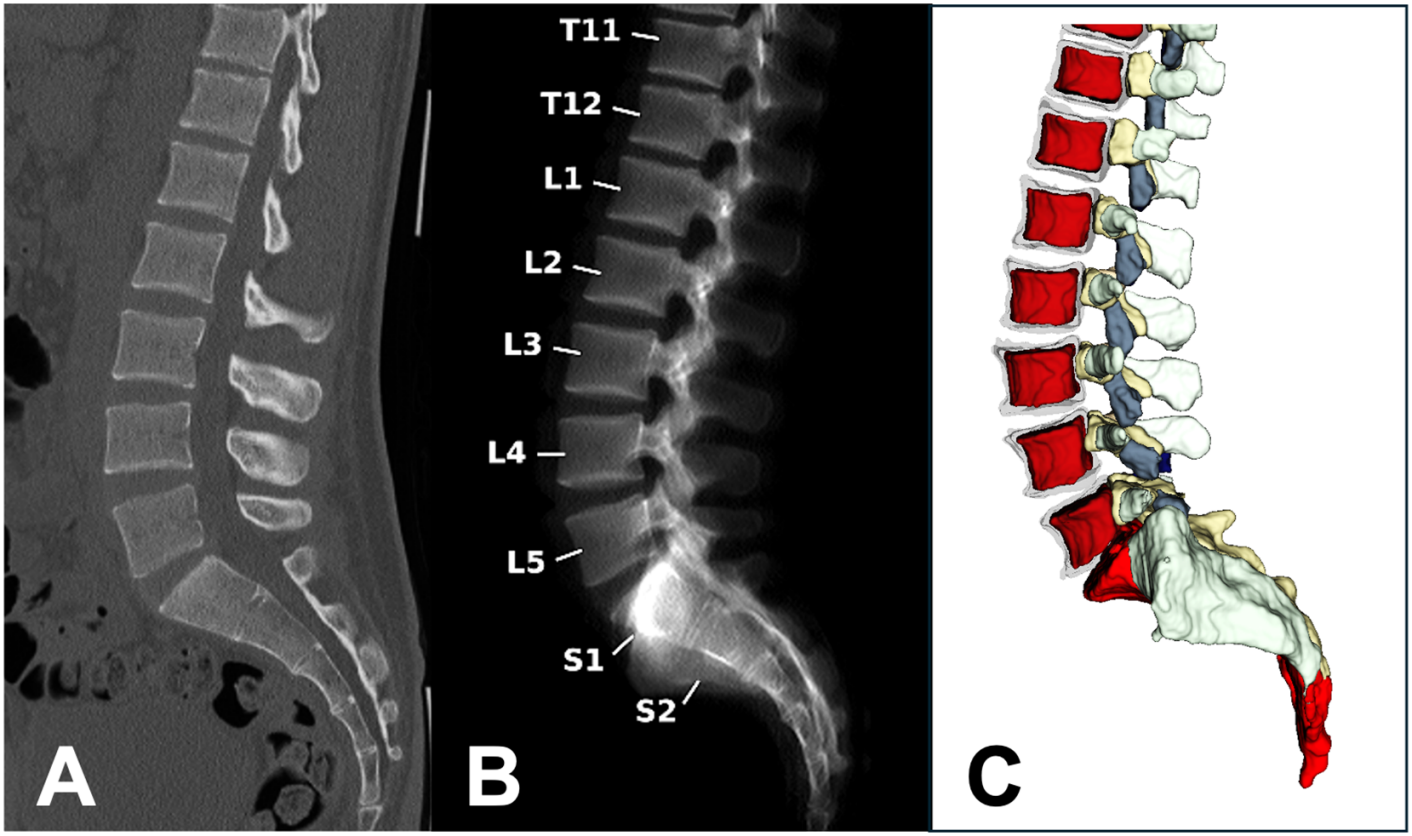
An exemplary case evaluated by SpineQ, showing a non-contrast enhanced CT scan of a 51-year-old female patient with a sagittal view of the thoracolumbar spine **(A)**. Automated vertebral body labeling was performed **(B)** for vBMD measurements with asynchronous calibration. Spine segmentation, including subregion segmentation, was performed, and masks of the trabecular compartment (red) were obtained **(C)**. Posterior elements and the sacrum were also routinely segmented (depicted in gray, yellow, and light red). For bone health evaluation, vBMD values of L1-3 were averaged, resulting in an average vBMD_L1-3_ = 104.9 mg/cm³ in this patient. CT, computed tomography; vBMD, volumetric bone mineral density.

Volumetric bone mineral density (vBMD) was assessed using a novel technique that employs routine computed tomography (CT) imaging data from the thoracolumbar spine, with measurements obtained via SpineQ software v1.0 (Bonescreen, Munich, Germany), a convolutional neural network (CNN)-based framework specifically developed for automated opportunistic vBMD assessment (**Figure 2**) (28–30). SpineQ software automates spinal processing, including allowing for separate characterization of the trabecular and cortical vertebral compartments. vBMD (expressed in mg/cm^3^) was extracted from the trabecular region in measurable vertebrae. Vertebrae were excluded from BMD measurements if they met any of the following criteria: (1) presence of any fracture, including those of malignant, traumatic, or osteoporotic origin; (2) severe degenerative changes such as sclerotic alterations of the endplates; (3) presence of hardware. All CT scanners were calibrated using asynchronous calibration with scanner-specific conversion factors to ensure consistency of vBMD values across different scanners and imaging protocols. This calibration method has previously demonstrated high reproducibility across various cross-session and cross-scanner settings (31). vBMD values were averaged over measurable lumbar vertebrae L1-L3 (32). If none of these vertebral levels could be assessed because of the exclusion criteria mentioned above, vBMD values were averaged over available vertebrae Th11-L5. All measurements were reviewed by an experienced radiologist and a neuroradiologist to ensure accurate recognition, labeling, and segmentation of the vertebrae.

### 2.6 Statistical analysis

The MACS group was identified and matched by age and sex with PA patients with biochemically excluded cosecretion (non-MACS group). Statistical analysis was performed using R statistical software (R version 3.6.3). Descriptive statistics were used to determine median and ranges depending on data distribution (parametric or non-parametric, respectively), while categorical data are shown as counts and percentages. Associations between variables were assessed using one-way ANOVA for continuous variables, and we adjusted for multiple comparisons using Bonferroni corrections. Spearman’s Rank Correlation Coefficient (rs) was used to measure the strength and direction of the monotonic relationship between cortisol after DST and vBMD based on their ranks. A paired t-test was used for group comparisons, as matching by age and sex introduced a level of dependency between the groups, and the distribution of differences between paired values approximated normality. P-values less than 0.05 were considered significant.

## 3 Results

### 3.1 Clinical characteristics

The included PA patients (total n = 100) had a median age of 60 years (54, 69) and were predominantly male (60%). Fifty patients with confirmed MACS were matched by age and sex with patients who had exclusion of MACS (non-MACS, range 0.1-1.7 µg/dL/2.8 - 46.9 nmol/L). Median cortisol after DST was 1.1 µg/dL (30.3 nmol/L) [IQR: 0.5 µg/dL (13.8 nmol/L)] in the non-MACS group and 2.5 µg/dL (69.0 nmol/L) [IQR: 1.4 µg/dL (38.5 nmol/L)] in the MACS group (p < 0.001). In the MACS group, none of the patients met the biochemical and clinical criteria for overt Cushing syndrome (33). There was no significant difference in median concentrations of triglycerides and cholesterol between the groups (**Table 1**, p= 0.5 and 0.06, respectively). Furthermore, there was no significant difference in smoking or reported alcohol consumption between the non-MACS and MACS groups. Forty-nine patients had a visible and measurable adrenal gland lesion on non-contrast-enhanced CT. By means of AVS, 38 patients had lateralized disease and 62 had bilateral disease, while there was no difference between MACS versus non-MACS regarding unilateral or bilateral disease (p = 0.7).

**Table 1:** Clinical and laboratory data.

Characteristic	MACS		p-value*^2^*
	No, n = 50*^1^*	Yes, n = 50*^1^*	
Age (years)	60 (52, 68)	60 (51, 67)	> 0.9
Sex			0.5
Male	30 (60%)	30 (60%)	
Height [meters]	1.76 (1.68, 1.82)	1.72 (1.65, 1.78)	0.2
BMI [kg/m2]	27.8 (25.3, 31.0)	28.5 (25.5, 31.8)	0.9
Triglycerides [mg/dL]³	92 (65, 131)	102 (66, 144)	0.5
Total cholesterol [mg/dL]³	180 (158, 205)	195 (174, 223)	0.06
HbA1c %	5.25 (5.00, 5.80)	5.30 (5.10, 5.60)	0.9
ACTH [pg/mL]³	11 (8, 16)	14 (8, 21)	0.3
Waist to hip ratio	0.96 (0.90, 1.02)	0.93 (0.84, 1.01)	0.5
Lateralization			0.7
Yes	18 (36%)	20 (40%)	
No	32 (64%)	30 (60%)	
Lesion on CT	21 (42%)	28 (56%)	0.2
Aldosterone baseline [ng/L]³	166 (131, 256)	206 (131, 286)	0.4
Renin [mU/L]³	3.2 (2.0, 6.6)	3.5 (2.0, 6.5)	0.9
Aldosterone 4h after SIT [ng/L]³	105 (84, 182)	113 (79, 200)	0.8
Direct Renin Concentration 4h after SIT [mU/L]³	2.00 (2.00, 3.90)	2.25 (2.00, 4.70)	0.8
Aldosterone-renin-ratio [ng/mU]³	46 (28, 79)	58 (36, 94)	0.3
Lowest level of potassium [mmol/L]	3.30 (3.03, 3.80)	3.44 (3.13, 3.80)	0.5
GFR [mL/min/1.73m2]	89 (77, 99)	84 (68, 96)	0.1
Creatinine [mg/dL]³	0.90 (0.80, 1.00)	0.90 (0.80, 1.10)	0.6
Sodium [mmol/L]	142 (140, 143)	141 (141, 143)	0.6
Calcium [mmol/L]	2.37 (2.30, 2.47)	2.38 (2.33, 2.42)	0.9
PTH [pg/mL]	67.3 (48.9, 85.1)	55.7 (47.4, 75.3)	0.2
Vitamin D [ng/mL]	19 (13, 25)	22 (16, 31)	0.3
Systolic 24h-BP [mmHg]	153 (144, 164)	155 (147, 169)	0.4
Diastolic 24h-BP [mmHg]	94 (84, 101)	92 (85, 103)	0.9
Defined daily dose (DDD)	1.50 (1.00, 2.94)	2.00 (0.75, 3.00)	0.3

*^1^* Median (IQR); n (%).

*^2^* Wilcoxon rank sum exact test; Wilcoxon rank sum test; Pearson’s Chi-squared test

24h-BP, 24-hour blood pressure; CT, computed tomography; GFR, glomerular ﬁltration rate; MACS, mild autonomous cortisol secretion; PTH, Parathyroid hormone; SIT, saline infusion test; SD, standard deviation.

³Parameters in SI units (MACS no; MACS yes): Triglycerides (1.0 mmol/l; 1.2 mmol/l), Total cholesterol (4.7 mmol/l; 5.1 mmol/l), ACTH (2.4 pmol/l; 3.1 pmol/l), Aldosterone baseline (460.5 pmol/l; 571.4 pmol/l), Aldosterone 4h after SIT (291.3 pmol/l; 313.5 pmol/l), Creatinine (79.6 µmol/l; 79.6 µmol/l).

The prevalence of cardiovascular diseases was comparable between the MACS and non-MACS groups, both at 48% (p = 1.0). Insufficiency fractures were documented in one patient from the MACS group and two from the non-MACS group (p= 0.7). The prevalence of T2DM was also similar, with 7 patients (14%) in the MACS group and 8 patients (16%) in the non-MACS group (p= 0.8). Prior to CT scans, vitamin D and calcium supplementation was recorded in 5 patients in the MACS group and 4 patients in the non-MACS group (p= 0.7). No patient was on chronic oral glucocorticoid or antiresorptive therapy, and there was no significant difference in statin or proton pump inhibitor use between the groups. Only two patients in the MACS group were taking antiepileptic drugs, and none were on estrogen therapy. Given this, the potential influence on false-positive DST results is considered minimal. Patients did not have a history of depression or alcoholism.

### 3.2 Bone turnover markers and vBMD

**Figure 3:**
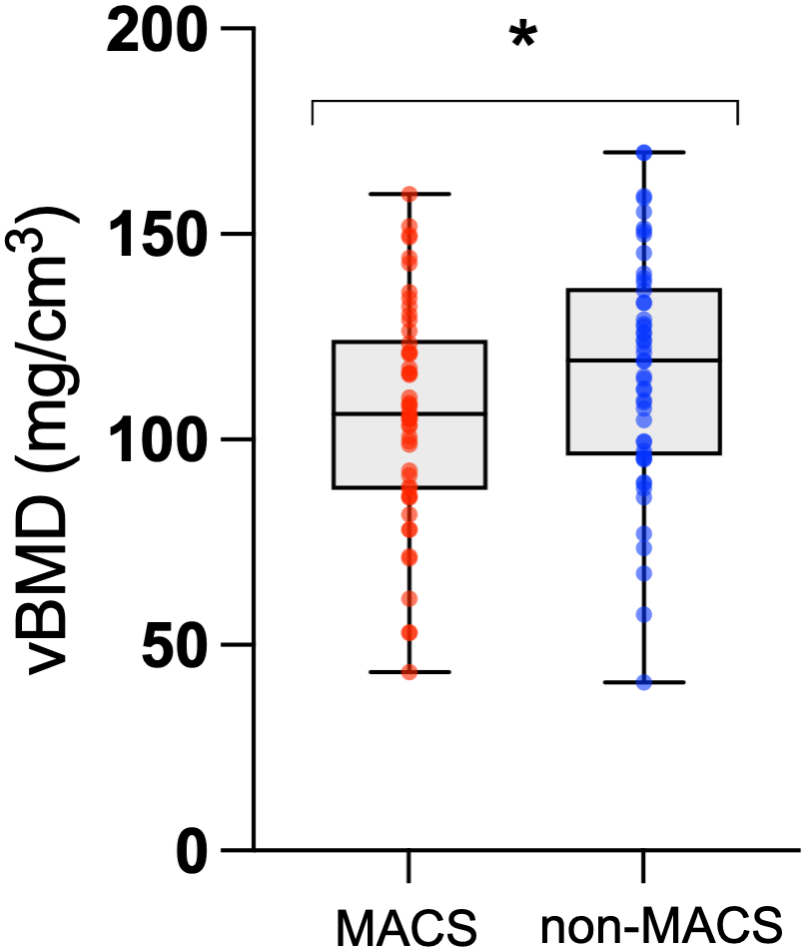
vBMD measurements in the MACS-group and in the non-MACS group. A paired t-test was used for comparison. *MACS-group vs. non-MACS group: p = 0.0380. Mean MACS-group: 106.4 mg/cm^3^. Mean non-MACS group: 116.6 mg/cm^3^. vBMD, volumetric bone mineral density.

**Figure 4:**
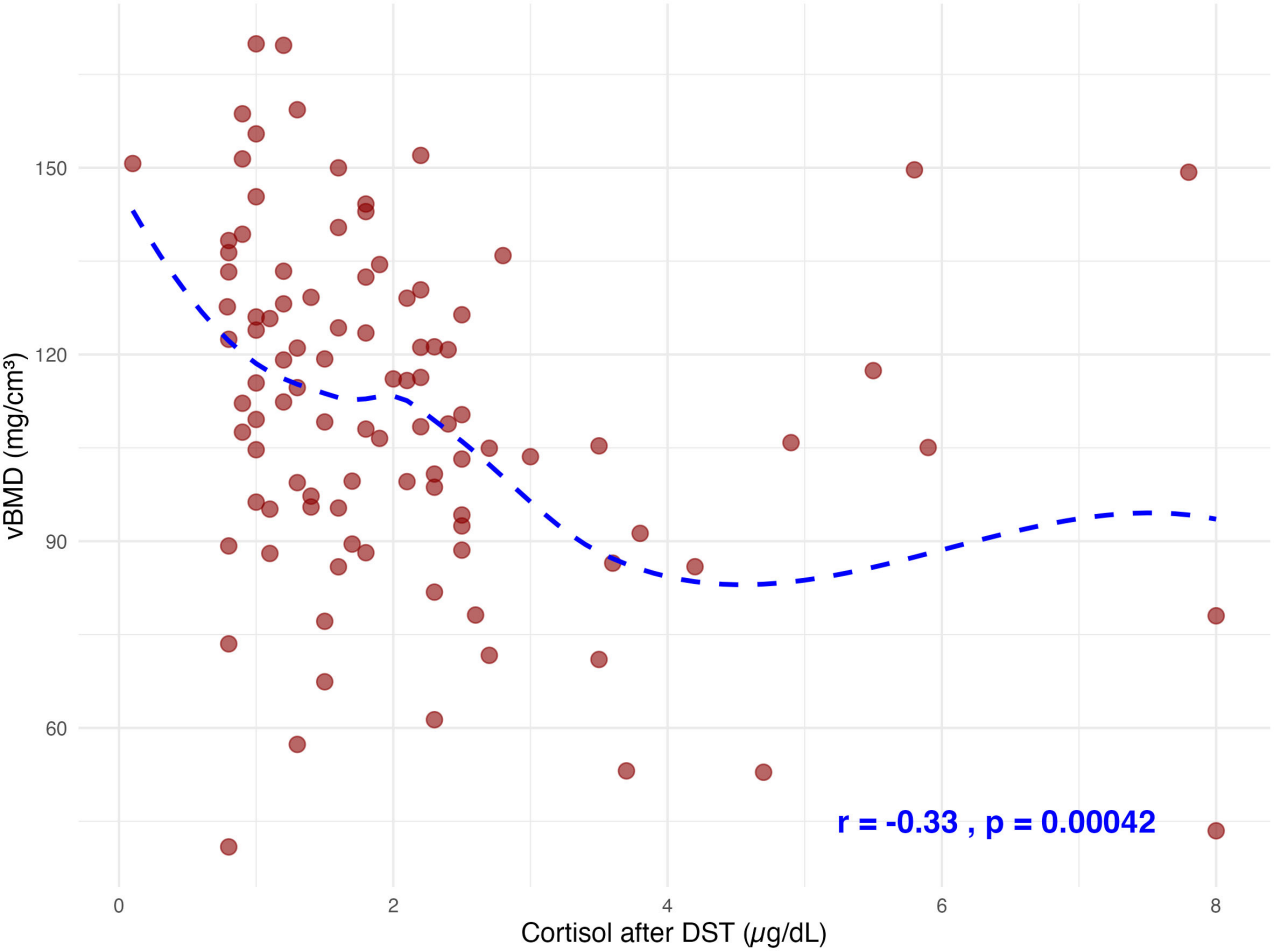
Spearman correlation analysis between cortisol levels DST and vBMD demonstrated a statistically significant negative association (Spearman’s r=−0.33, p=0.00042). Data points represent individual observations, with a fitted loess curve illustrating the trend across the dataset. DST, dexamethasone suppression test; vBMD, volumetric bone mineral density.

There were no statistically significant differences in the concentrations of bone turnover markers, including BAP, PINP, OC, and CTX-I, between MACS and non-MACS patients (**Table 2**; p = 0.4, 0.9, 0.3, and 0.09, respectively). Additionally, no significant differences were observed in median vitamin D (p = 0.3), corrected calcium (p = 0.9), and parathyroid hormone (PTH; p = 0.2). Cohort classifications based on visible lesions identified via CT or PA lateralization (unilateral vs. bilateral disease) through AVS also did not yield significant differences in those markers. In contrast, measurements of vBMD at the lumbar spine obtained from non-contrast enhanced CT imaging revealed a significantly lower mean vBMD in the MACS group compared to the non-MACS group (106.4 mg/cm³ vs. 116.6 mg/cm³; p = 0.04) (**Figure 3**). Furthermore, post-DST cortisol exhibited a significant negative correlation with vBMD (Spearman’s r = −0.33, p = 0.00042), as illustrated in **Figure 4**.

**Table 2:** Bone Turnover Markers.

Characteristic	MACS		
	No, n = 50*^1^*	Yes, n = 50*^1^*	p-value*^2^*
BAP [µg/L]	15.6 (13.2, 19.1)	15.4 (13.3, 19.0)	0.4
Osteocalcin [ng/mL]	17 (13, 21)	16 (14, 19)	0.3
PINP [ng/mL]	54 (48, 72)	59 (40, 73)	0.9
CTX-I [ng/mL]	0.32 (0.21, 0.45)	0.25 (0.16, 0.39)	0.09
Characteristic	Adrenal lesion in CT		
	No, n = 51*^1^*	Yes, n = 49*^1^*	p-value*^2^*
BAP [µg/L]	15.1 (13.2, 18.8)	16.0 (13.3, 19.3)	0.6
Osteocalcin [ng/mL]	17 (14, 21)	16 (13, 20)	0.5
PINP [ng/mL]	56 (42, 75)	56 (48, 72)	>0.9
CTX-I [ng/mL]	0.27 (0.18, 0.36)	0.31 (0.20, 0.45)	0.2
Characteristic	Lateralization in AVS		
	No, n = 62*^1^*	Yes, n = 38*^1^*	p-value*^2^*
BAP [µg/L]	15.2 (13.1, 18.6)	15.7 (13.7, 19.8)	0.3
Osteocalcin [ng/mL]	17 (14, 20)	16 (13, 20)	0.8
PINP [ng/mL]	54 (39, 73)	56 (50, 74)	0.3
CTX-I [ng/mL]	0.27 (0.18, 0.36)	0.36 (0.21, 0.48)	0.14

*^1^* Median (IQR); n (%).

*^2^* Wilcoxon rank sum exact test; Wilcoxon rank sum test; Pearson’s Chi-squared test

AVS, adrenal venous sampling; BAP, bone-speciﬁc alkaline phosphatase; CT, computed tomography; CTX-I, Carboxy-terminal crosslinked telopeptide of type I collagen; MACS, mild autonomous cortisol secretion; PINP, N-terminal propeptide of type 1 collagen. MACS was confirmed by 1-mg DST with a cutoff of ≥ 1.8 µg/dl.

A lateralization index for AVS greater than 4.0, or a lateralization index between 3 and 4 together with a contralateral index below 1.0 were considered to be compatible with unilateral disease.

## 4 Discussion

To our knowledge, this is the first study to compare bone turnover biomarkers and vBMD in patients diagnosed with PA and concomitant MACS. We found that the only significant distinguishing marker for MACS in PA patients was the vBMD of the lumbar vertebrae, while the bone turnover markers did not differ significantly between groups with and without MACS. Notably, vBMD exhibited a significant negative correlation with serum cortisol levels following 1 mg DST.

A recent large multicenter study indicated a positive correlation between MACS and an increased risk of vertebral fractures, suggesting that chronic cortisol exposure may impair the bone health (34). While the mechanisms through which glucocorticoids affect bone are well-established, the role of aldosterone in bone metabolism is not fully understood. Emerging studies suggest that patients with PA may experience bone alterations; however, the results are heterogeneous, and data regarding BMD and fracture risk are limited (18, 35). Given that all patients in our study had PA, we cannot discount the possibility that elevated aldosterone levels may have influenced bone quality. The absence of significant differences in bone turnover markers between the two groups is consistent with previous research comparing MACS patients to those with NFATs (18). However, in patients with NFATs, even slightly elevated glucocorticoid concentrations, which are not sufficient to cause typical Cushing’s symptoms, can pose a significant risk by decreasing bone mineral density and compromising bone quality, thereby increasing the likelihood of vertebral fractures (36, 37). Although a possible effect of aldosterone in bone would be possible, the data reinforces the stronger effect of the combination of aldosterone and cortisol excess on bone health. Our study found no significant differences in bone turnover markers based on lesion classification or lateralization, further supporting the idea that cortisol excess has a more pronounced effect on bone metabolism than aldosterone excess, even in the potentially more severe unilateral primary aldosteronism.

The criteria for diagnosing mild hypercortisolism have undergone significant changes in recent years. The European Society of Endocrinology (ESE) in collaboration with the European Network for the Study of Adrenal Tumors (ENSAT) recently released new guidelines for the diagnosis of mild hypercortisolism, now defined by the term mild autonomous cortisol secretion (MACS) (9). According to these guidelines, the diagnosis of MACS in patients with adrenal incidentalomas (AI) should be based on serum cortisol levels exceeding 50 nmol/L (1.8 µg/dL) after a 1 mg DST, in the absence of classical overt Cushing syndrome symptoms. Consequently, all patients with MACS should be screened for potential cortisol-related comorbidities to ensure appropriate therapeutic interventions.

The reported prevalence of PA among AI is between 2% and 5% (38, 39). Although excess glucocorticoid secretion in primary aldosteronism was previously regarded as rare, recent studies indicate its commonality and a strong association with increased metabolic risk, challenging the traditional distinctions between Cushing’s and Conn’s syndromes (40). This highlights the importance of screening for mild cortisol secretion as a preventative measure. The rationale is that by early identification and therapeutic management of mild cortisol secretion, it may be possible to treat bone disease earlier and therefore reduce the risk of long-term bone alterations and associated complications, such as fractures. However, further studies are necessary to better investigate the indication for specific antiresorptive or anabolic therapy in patients with primary aldosteronism and MACS, and whether the same criteria for deciding such therapy (such as the FRAX score and risk of future fractures) would be applicable. While CT scans expose patients to ionizing radiation, standardized CT-based vBMD analysis can be opportunistically performed using non-contrast CT imaging obtained from routine clinical workups for PA or incidental adrenal findings, as demonstrated in this study. This novel approach, utilizing a CNN-based framework, may assist in stratifying PA and MACS patients for glucocorticoid-opposing treatments, thereby mitigating metabolic risks, preserving bone health, and reducing fracture rates.

## 5 Strengths and limitations

A clear strength of this study is the 1:1 matching of cohorts of PA patients with and without MACS and the standardized automated analyses of vBMD in non-contrast enhanced CT imaging of the lumbar vertebrae. Furthermore, the robustness of vBMD measurement has been consistently validated in direct comparisons with dedicated quantitative CT (QCT), with SpineQ demonstrating high diagnostic accuracy across scanner types (ICC: 0.913) and strong fracture discrimination (AUC: 0.86), outperforming QCT in distinguishing patients with osteoporotic vertebral fractures (AUC: 0.81) (28).

A limitation of our study is the absence of a NFAT group for comparison with patients without hormonal secretion abnormalities, which could have clarified the isolated effects of aldosterone excess. The retrospective nature of our analysis restricted access to comprehensive data on NFAT patients, who do not consistently undergo the extensive diagnostic work-up received by PA patients. This inconsistency limits our ability to make effective comparisons between the two groups. Another possible limitation is that due to the retrospective nature of this analysis, it is difficult to exclude altered cortisol results due to pseudo-Cushing’s syndrome. However, there was no documentation of alcoholism or depression in the medical history and the patients were not obese.

## 6 Conclusion

MACS in PA patients significantly reduces bone density, a change that is not reflected in the levels of bone turnover markers. This finding highlights the importance of recognizing the impact of MACS on skeletal health in this population. Moreover, this novel approach suggests that CT scans, which are an integral part of the adrenal mass screening process, can be opportunistically utilized for the early detection of alterations in bone density among PA patients with MACS. Such early detection may provide critical insights for managing the long-term bone health of these individuals.

## Data Availability

The raw data supporting the conclusions of this article will be made available by the authors, without undue reservation.
